# A Modified Sol–Gel Synthesis of Anatase {001}-TiO_2_/Au Hybrid Nanocomposites for Enhanced Photodegradation of Organic Contaminants

**DOI:** 10.3390/gels8110728

**Published:** 2022-11-10

**Authors:** Abubakar Katsina Usman, Diana-Luciana Cursaru, Gheorghe Brănoiu, Raluca Şomoghi, Ana-Maria Manta, Dănuţa Matei, Sonia Mihai

**Affiliations:** 1 Faculty of Petroleum Technology and Petrochemistry, Petroleum-Gas University of Ploiesti, 100680 Ploiești, Romania; 2 Department of Pure and Industrial Chemistry, Bayero University Kano, PMB 3011, Kano 70006, Nigeria; 3 Faculty of Petroleum and Gas Engineering, Petroleum-Gas University of Ploiesti, 100680 Ploiești, Romania; 4 National Institute for Research and Development in Chemistry and Petrochemistry—ICECHIM, 060021 Bucharest, Romania

**Keywords:** modified sol–gel synthesis, PhACs, nanocomposites, capping agents, anatase TiO_2_

## Abstract

A sol–gel synthesis technique was employed for the preparation of anatase phase {001}-TiO_2_/Au hybrid nanocomposites (NCs). The scalable, schematic, and cost-efficient method was successfully modified using HF and NH_4_OH capping agents. The photocatalytic activity of the as-synthesized {001}-TiO_2_/Au NCs were tested over 2-cycle degradation of methylene blue (MB) dye and pharmaceutical active compounds (PhACs) of ibuprofen and naproxen under direct sunlight illumination at 35 °C and 44,000 lx. Transmission electron microscopy (TEM), high resolution transmission electron microscopy (HR-TEM), fast Fourier transform (FFT), X-ray diffraction (XRD), X-ray photoelectron spectroscopy (XPS), energy dispersive X-ray spectroscopy (EDS), and ultraviolet–visible diffuse reflectance spectroscopy (UV–Vis DRS) were employed for the characterization of the as-prepared sample. The characterization results from the TEM, XPS, and XRD studies established both the distribution of Au colloids on the surface of TiO_2_ material, and the presence of the highly crystalline structure of anatase {001}-TiO_2_/Au NCs. Photodegradation results from the visible light irradiation of MB indicate an enhanced photocatalytic performance of Au/TiO_2_ NCs over TiO_2_. The results from the photocatalytic activity test performed under direct sunlight exposure exhibited promising photodegradation efficiencies. In the first cycle, the sol–gel synthesized material exhibited relatively better efficiencies (91%) with the MB dye and ibuprofen, while the highest degradation efficiency for the second cycle was 79% for the MB dye. Pseudo first-order photodegradation rates from the first cycle were determined to be comparatively slower than those from the second degradation cycle.

## 1. Introduction

The global replenishment of organic dyes and pharmaceutical active compounds (PhACs) into the environment exacerbates the ecological threats posed by the process and the resultant effluent pollutants to the aquatic system. These organic contaminants get into water through diverse activities of the pharmaceutical, textile, and food industries. Many studies have forewarned of the serious risks associated with these pollutants to human health, even in small traceable quantities. Data show that between 60 and 80% of PhACs are not being absorbed by humans or animals [1]; this signifies that thousands of tons of PhACs are being discharged into the environment on a daily basis. For optimum pollution control of PhACs, their sources must be targeted.

Several approaches have been employed to tackle the water pollution caused by organic dyes and pollutants of emerging concern such as PhACs. These include electrochemical methods that employ different electrode materials [2], and biological remediation such as phytoremediation or the bioreactor approach [3]. Others are ozonation and advanced oxidation [4,5] as well as photo-assisted catalysis [6]. However, conventional wastewater treatment technologies such as adsorption and biological processes are challenged by the formation of harmful bi-products, and have been extensively reported to be deficient in the complete removal of these contaminants [7,8]. This necessitates the substantial efforts being made by many researchers to design an optimal purification technique for the successful elimination of these obstinate contaminants from the surface water and groundwater.

Photo-induced heterogeneous nanocatalysis—a proficient advanced oxidation process (AOP)—has been greatly used for the removal of these recalcitrant organic pollutants from wastewater [9]. Titanium dioxide (TiO_2_) serves as the researchers’ paradigm of ideal photocatalytic materials, with anatase and rutile crystal structures the two most common forms of its polymorphs [10]. The anatase TiO_2_ has been widely reported as the more reactive phase than the rutile. Luttrell et al. [11] reported that the bulk transfer of excitons to the anatase surface contributes to its greater surface reaction than in rutile. Another parameter that strongly determines the reactivity and photocatalytic performance of the TiO_2_ is its orientation, with {001} facets proven to be more reactive than the other facets due to their greater degree of reduction and low density surface under well-layered Ti atoms [12]. However, the two main unresolved drawbacks that retard the performance of TiO_2_ remain its limited light application to UV range, and the fast recombination of charge carriers.

The deposition of plasmonic metal nanoparticles (NPs) such as Pt-NPs, Ag-NPs, and Au-NPs on metal oxide photocatalysts increases the photo-current density of the nanosheets and acts as a plasmon source in the visible light region [13]. This technique is among the most promising to extend its light absorption. Recently, precious metals of Au, Ag, Pd, and Pt were used by Conte et al. [14] to modify the TiO_2_ surface for renewable energy applications. The plasmon effect and optoelectronic properties of the metals as well as their function as an electron sink were principally determined to enhance the performance of TiO_2_ through band gap reduction. In 2021, a platinized surface of {001}-TiO_2_ NSs was successfully fabricated via the uniform dispersion of Pt colloids on TiO_2_ nanosheets by Zhao et al. [15]. Pt colloids facilitated hydrogen reduction of the reactive {001} orientations on TiO_2_ at 673 K.

Furthermore, various studies have demonstrated that the synthesis techniques employed in the fabrication of semiconductor-based photocatalysts largely determines their physicochemical properties and photocatalytic activity. Solution-phase methods such as sol–gel, hydrothermal/solvothermal, and co-precipitation are the most promising synthesis routes for photocatalysts because they are simple, cost-effective, and do not require complex equipment [16]. They also allow for the incorporation of capping agents that control the material growth and agglomeration. Among them, the sol–gel method is favorable because it allows for easy regulation of important variables such as size, shape, surface area, and the pore volume of the material at relatively lower process temperature and pressure [17]. Chebil et al. (2015) successfully deposited high quality ZnO on a porous, etched silicon with a preferential (100) orientation via the sol–gel spin coating method [18]. The technique aided in the attainment of porous Si, which showed better ZnO deposition than that deposited on the Si substrates.

The sol–gel synthesis method is one of the most potent wet-chemical methods for the preparation of nano-sized-metal-oxide semiconductor photocatalysts. The technique involves the formation of colloidal suspension (sol) and a semi-rigid colloidal network (gel) in two principal processing steps viz. the hydrolysis of metal oxide precursors to form the sol; and poly-condensation of the hydrolyzed sol solution into a gel-like material. Aging, drying, and calcination (thermal treatment) of the gel are other steps before the purified form of the desired material is obtained. Some of the important benefits of using the sol–gel synthesis method includes the formation of high purity composite materials, narrow particle size distribution, and a lower processing temperature requirement. The use of capping agents such as hydrogen fluoride (HF) and ammonium hydroxide (NH_4_OH) play a significant role in the control and stabilization of material growth. Previous studies using other wet chemical methods have revealed how the presence of fluoride (F^−^) ions on the TiO_2_ surface during preparation can influence the formation of the anatase phase TiO_2_ with a {001} orientation. For example, selective growth of {001} facets of anatase TiO_2_ was obtained by Yasir et al. in the presence of the HF capping agent using the solvothermal method [19].

**Figure 1:**
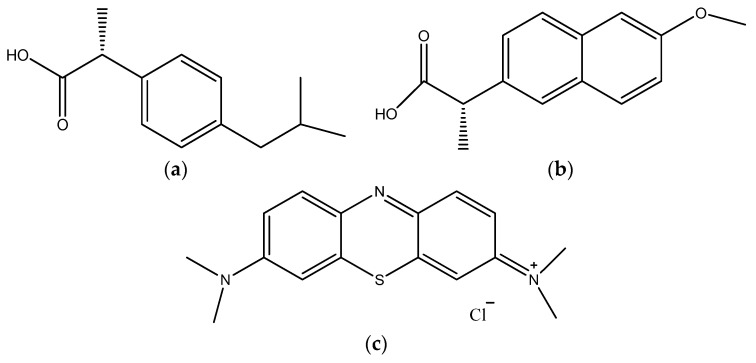
(**a**) Ibuprofen; (**b**) Naproxen; (**c**) Methylene blue dye.

In this work, degradation of PhACs of ibuprofen (Figure 1a) and naproxen (Figure 1b) as well as cationic MB (Figure 1c) under sunlight was achieved using {001}-faceted anatase-phase-TiO_2_/Au hybrid nanocomposites (NCs) prepared by the modified sol–gel method (MSGM) with HF and NH_4_OH as stabilizing agents. Evidence of improved photocatalytic performance of the nanocomposites was investigated using degradation of aqueous MB under controlled visible light irradiation.

## 2. Results and Discussion

### 2.1. X-ray Diffraction (XRD)

**Figure 2:**
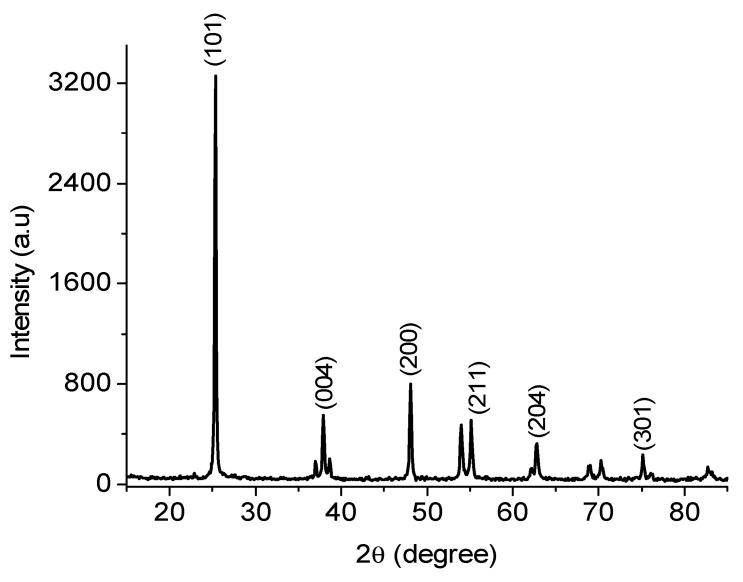
X-ray diffraction (XRD) patterns of the hybrid Au/TiO_2_ nanosheets.

In Figure 2, the presence of sharp diffraction peaks at 2θ values of 25.4, 37.9, 48.1, 53.2, 55.1, 62.8, and 75° could be observed. These peaks corresponded to the respective plane values (101), (004), (200), (105), (211), (204), and (301), which firmly indicates the formation of a crystalline structure that can be identified with anatase, a polymorph phase of TiO_2_ [20]. Exposure of the pure anatase phase surface of TiO_2_ can be established by the non-appearance of diffraction peaks at 2θ values of 27° and 31° for the rutile and brookite phases, respectively [21]. Preferred crystal growth along the (001) facet is indicated by the appearance of a broad (004) diffraction peak at 2θ = 37.9° [22]. Diffraction peaks associated with the gold colloids could not be observed. This may either be due to the possibility of overlapping between the diffractograms of TiO_2_ and gold colloids, or that the equipment was unable to detect the signals corresponding to gold colloids, which can be attributed to low Au-NP loading [23]. The refinement of the crystal structure by the Rietveld method of anatase TiO_2_ with body-centered tetragonal symmetry belonging to the space group I41/amd (no. 141), the following parameters of the crystal structure were obtained: a = b = 3.760 Å and c = 9.494 Å. The refinement of the anatase crystal structure also allowed for the determination of the interplanar distances for the main planes of its crystal structure: (101) d = 3.512 Å; (004) d = 2.373 Å; (200) d = 1.890 Å; (105) d = 1.697 Å; (211) d = 1.664 Å; (204) d = 1.478 Å; (220) d = 3.336 Å; (215) d = 1.262 Å. In the anatase crystal structure, the bond length of the Ti–O connections is in the range of 1909–1946 Å; Ti–Ti is 3034 Å; other interatomic distances being those between oxygen atoms O–O with a length between 2378–3034 Å.

### 2.2. X-ray Photoemission Spectroscopy (XPS)

**Figure 3:**
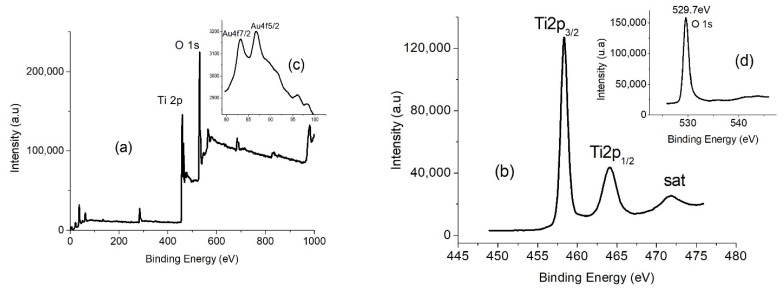
X-ray photoemission spectroscopy (XPS) peaks of (**a**) convoluted spectra of Ti 2p, O 1s, and Au 4f; (**b**) deconvoluted Ti 2p spectra; (**c**) deconvoluted Au 4f spectra; and (**d**) deconvoluted O1s spectra.

To further ascertain the electronic structure and chemical state of the as-synthesized material, XPS analysis was carried out, and it revealed clear, convoluted spectra (Figure 3a) of the Ti, Au, and O elements. Upon deconvolution of the Ti (2p) peak (Figure 3b) from the XPS spectrum, peaks corresponding to the binding energies (BE) of Ti were observed at the 458.42 and 464.08 eV states, which perfectly fit the Ti (2p_3/2_) and Ti (2p_1/2_) binding energy characteristics [24]. The splitting energy of 5.66 eV confirms the presence of the Ti^4+^ oxidation state between the two peaks [25]. The BE of 529.70 eV corresponding to O (1S) from the deconvoluted O (1s) XPS spectrum (Figure 3d) confirms the Ti–O–Ti chemical bonding in the synthesized material [26]. The decomposed XPS spectrum of Au (4f) (Figure 3c) was also studied to establish the presence of Au colloids in the synthesized nanosheets. The chemical state of Au in Au/{001}TiO_2_ NSs gave rise to two peaks at 83.17 eV and 86.75 eV corresponding to the Au (4f_7/2_) and Au (4f_5/2_) core-level excitations. The small deviation in the Au (4f_7/2_) BE peak position (83.17 eV) relative to the Au-bulk standard theoretical value of 84 eV indicates the successful preparation of Au-NPs [27]. The spin-orbit coupling of 3.58 eV between the Au (4f_7/2_) and Au (4f_5/2_) peaks validates the retention of the Au^0^ metallic character [28].

### 2.3. Transmission Electron Microscopy-Energy Dispersive X-ray (TEM-EDS)

**Figure 4:**
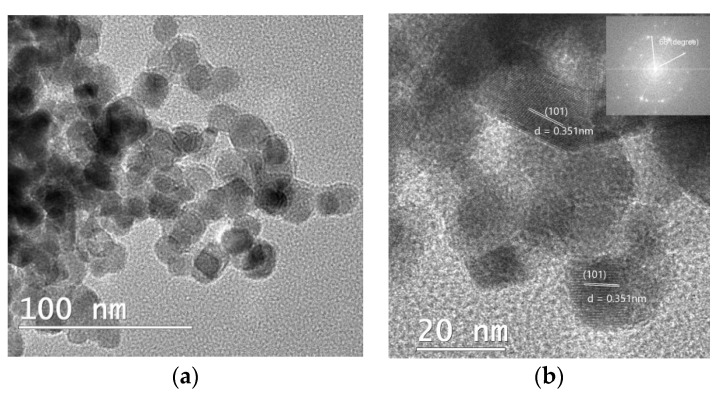

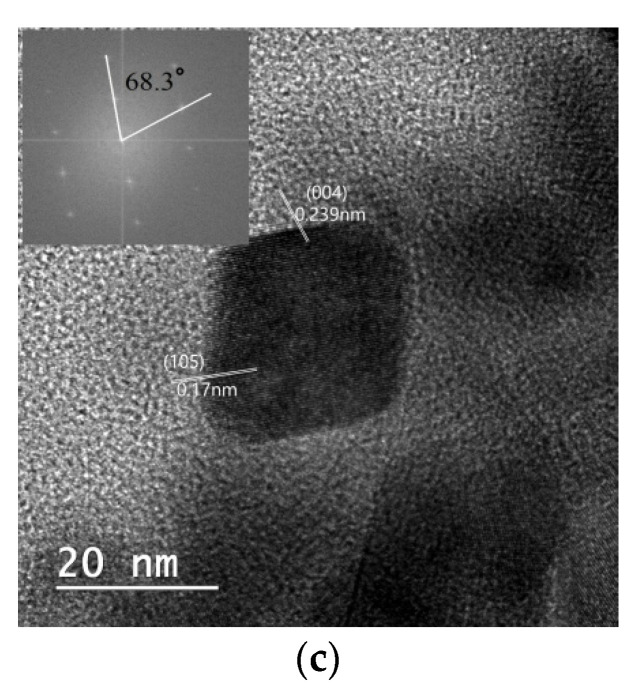
(**a**) TEM image of TiO_2_ NSs; (**b**,**c**) HR-TEM images obtained from a small portion of (**a**); insert HR-TEM corresponding FFT image.

**Figure 5:**
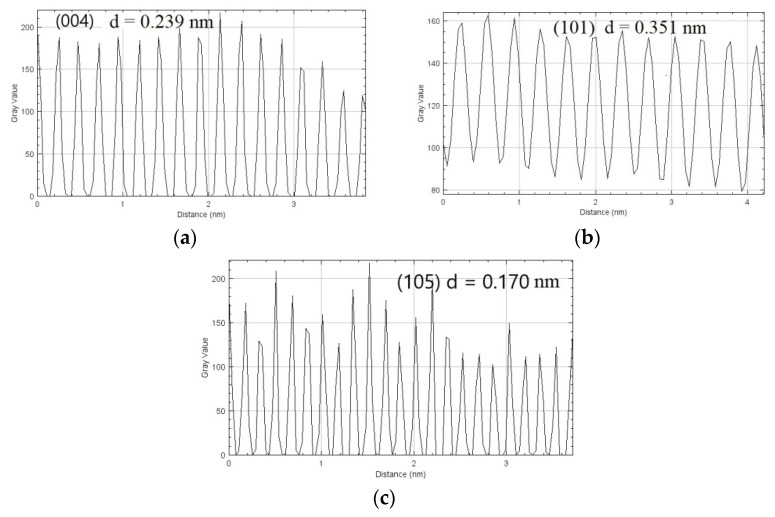
Magnified lattice fringes for d-spacing calculation in (**a**) (004), (**b**) (101), and (**c**) (105) peaks.

**Figure 6:**
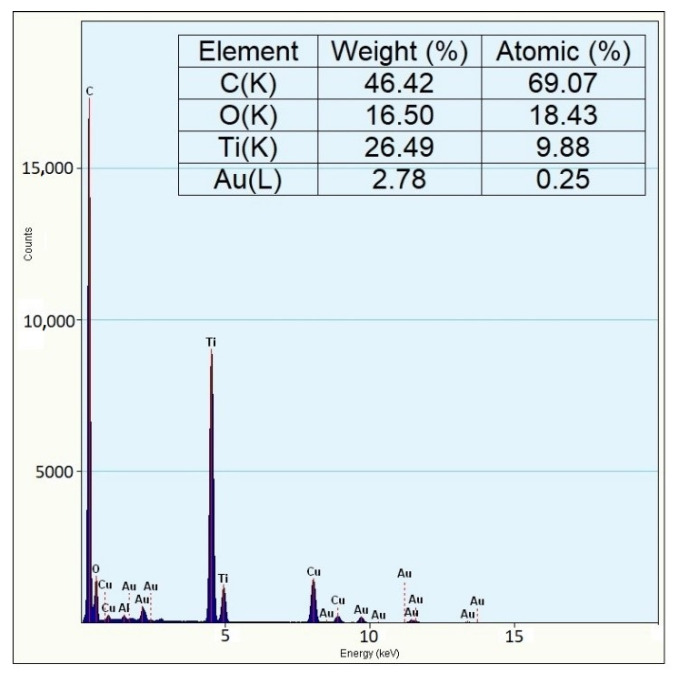
Energy dispersive X-ray (EDX) image for the as-synthesized TiO_2_/Au NSs.

The TEM and EDS images further indicate the prospect of Au-NPs hybridization in the accessible TiO_2_ sites with a size range between 20 and 25 nm. The TEM image (Figure 4a) clearly displays near ellipsoid shape particles, with darker particles randomly distributed on the TiO_2_ surface assumed to be the Au-NPs. The polycrystalline nature and d-spacing of the as-synthesized photocatalyst were further revealed by the complementary information from HR-TEM (Figure 4b,c) and its corresponding FFT (Figure 4d) analysis using Image J software. FFT analysis of the noise-refined TEM lattice fringe images (Figure 5a–c) revealed lattice spacing of 2.39 Å and 3.51 Å with an interfacial angle of 68.3° between them, which is consistent with the theoretical characteristics of the (004) and (101) lattice planes [29]. The supplementary data from EDS (Figure 6) examination confirms the presence of Ti, Au, and O, which corroborates the XPS data, and confirms the assumption from the TEM analysis that Au-NPs are randomly distributed on the surface of TiO_2_ NCs.

### 2.4. Ultraviolet–Visible Diffuse Reflectance Spectroscopy (UV–Vis DRS)

**Figure 7:**
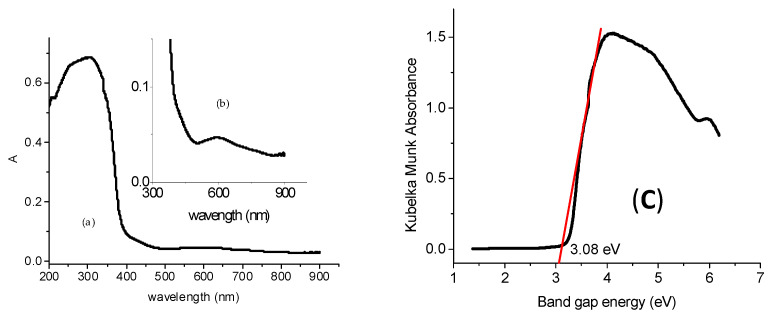
(**a**) UV–Vis diffuse absorption spectra of TiO_2_/Au; and (**b**) Additional peak shoulder in the visible region; and (**c**) Tauc plot for band gap determination of TiO_2_/Au NSs.

The band gap of the as-prepared NCs was determined by the Tauc plot from the Kubelka–Munk absorbance obtained from the UV–Vis diffuse absorption spectra. The UV–Vis diffuse absorption spectra (Figure 7a) of TiO_2_/Au NSs displayed a strong absorption peak shoulder in the UV region at ~384 nm, and another peak shoulder in the visible region at ~592 nm, which corresponded to band gap energies of 3.23 eV for TiO_2_ and 2.09 eV for Au-NPs. The Tauc plot (Figure 7b) shows a decrease in the band gap energy from 3.23 eV to 3.08 eV, thanks to the deposited Au-NPs in the TiO_2_ lattice, which results in the light red shift from the UV to visible region that is believed to enhance photocatalytic activity.

### 2.5. Photocatalytic Activity

In order to examine the photocatalytic performance of TiO_2_ and Au/TiO_2_ NCs, visible light degradation of MB and solar-assisted degradation of MB, ibuprofen, and naproxen were performed.

**Figure 8:**
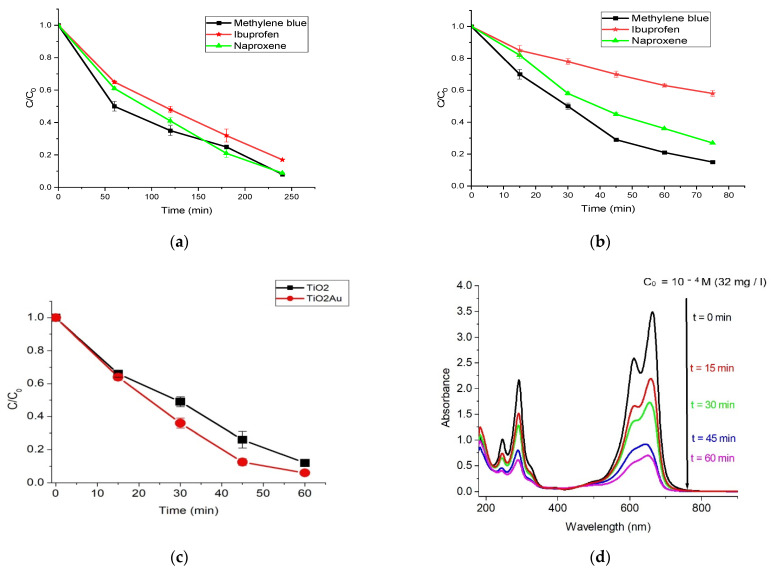

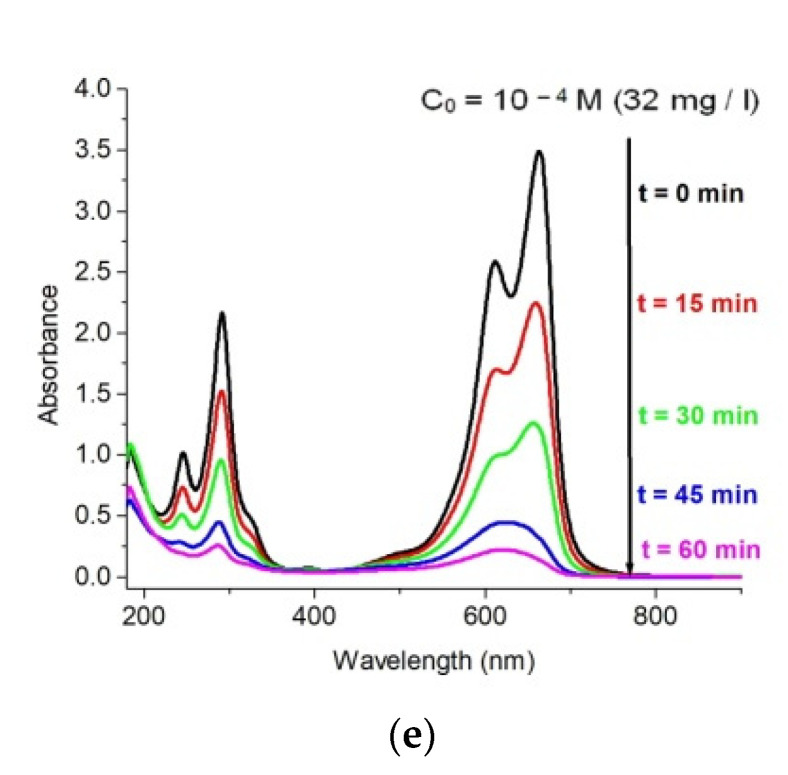
Photocatalytic degradation of methylene blue (MB) dye, ibuprofen, and naproxen for (**a**) cycle 1 and (**b**) cycle 2 under direct solar irradiation; (**c**) degradation of MB under controlled visible light irradiation; and UV–visible spectra of (**d**) TiO_2_ and (**e**) Au/TiO_2_ NCs for MB degradation.

Figure 8d,e show the UV–visible absorption spectra of MB degradation by pristine TiO_2_ and Au/TiO_2_ NCs, respectively, using a controlled visible light illumination. It can be seen that the maximum absorption peak was around 665 nm, which corresponds to the characteristic peak of MB. The reduction in the absorption peaks with an extension in the irradiation time signifies the progressive degradation of MB. Under visible light irradiation, both pristine TiO_2_ and Au/TiO_2_ NCs were able to remarkably degrade MB, with Au/TiO_2_ NCs having superior photocatalytic performance over pristine TiO_2_. For instance, after 60 min of controlled visible light irradiation, Au/{001}-TiO_2_ NCs exhibited 93.71% photodegradation efficiency compared to the 87.14% efficiency of the pristine TiO_2_.

The photocatalytic activity test of TiO_2_/Au NCs was also carried out for mineralization of MB dye, ibuprofen, and naproxen in two photodegradation cycles under direct sunlight exposure. No significant mineralization was recorded for all three contaminants when the reaction was conducted in the absence of the catalyst after 2 h.

**Figure 9:**
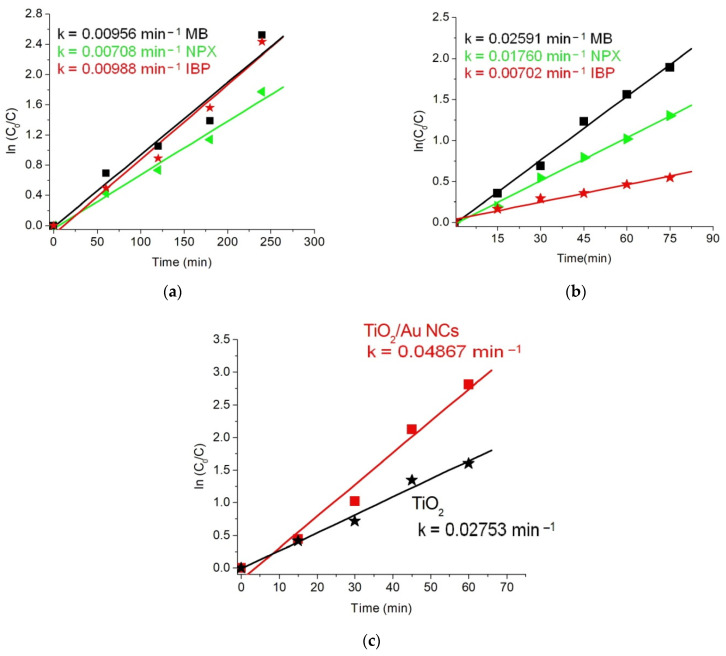
Pseudo-first order kinetics of (MB) dye, ibuprofen, and naproxen for (**a**) cycle 1 and (**b**) cycle 2 under direct sunlight exposure; and (**c**) MB under controlled visible light irradiation.

The photodegradation efficiencies for the two photodegradation cycles are presented in Table 1, with their corresponding fitting results in Figure 8a for cycle 1 and Figure 8b for cycle 2. The results from the first cycle show that naproxen had a lower degradation efficiency of about 83% than both MB and ibuprofen, with about 92% each after 4 h. However, results from cycle 2 suggest a higher degradation rate within the first 60 min than cycle 1, which can be seen from the pseudo first-order plots (Figure 9a,b). This may be connected to an improved adsorption capacity of the sample photocatalyst after cycle 1.

### 2.6. Proposed Mechanism

The proposed reaction mechanism based on the photocatalytic reactions data are explained in the following equations [30,31]. In summary, photo-excited charge carriers are formed upon strong absorption of the ultraviolet A (UVA) region of the solar irradiation by {001}-TiO_2_/AuNSs.

(1) $$\mathrm{Au}/{\mathrm{TiO}}_{2}\left\{001\right\}\overset{\mathrm{hv}\;\left(\mathrm{solar}\right)}{\Rightarrow }\mathrm{Au}/{\mathrm{TiO}}_{2}\left\{001\right\}\left(e_{\mathrm{CB}}^{-}+h_{\mathrm{VB}}^{+}\right)$$

The strong absorption within the visible region was aided by the presence of the distributed Au colloids on the TiO_2_ surface. The photo-induced holes in the VB of TiO_2_ then migrate to the catalyst surface to react with the adsorbed hydroxyl (${\mathrm{OH}}_{\mathrm{adsorbed}}^{-}$) ions on the catalyst surface, generating strong oxidizing $●{\mathrm{OH}}_{\mathrm{adsorbed}}$ radicals.

(2) $${\mathrm{OH}}_{\mathrm{adsorbed}}^{-}+h_{\mathrm{VB}}^{+}\to ●{\mathrm{OH}}_{\mathrm{adsorbed}}$$

The photo-generated electrons in the conduction band of the catalyst combine with O_2_ to form $●O_{2}^{-}$ radicals, as follows:

(3) $$O_{2}+e_{\mathrm{CB}}^{-}\to ●O_{2}^{-}$$

The highly oxidized radicals ($●{\mathrm{OH}}_{\mathrm{adsorbed}}\;\mathrm{and}\;●O_{2}^{-}$) produced in Equations (2) and (3) above can both oxidize the organic contaminants (MB dye, IBP, and NPX) coming into contact with the catalyst surface. Equations (4)–(6) below represent the degradation reactions of MB dye, naproxen, and ibuprofen with $●{\mathrm{OH}}_{\mathrm{adsorbed}}$ radicals. The corresponding reactions using $●O_{2}^{-}$ radicals are displayed in Equations (7)–(9). The photo-oxidation reactions pass through reaction intermediates that form CO_2_ and H_2_O on further oxidation reactions.

(4) $$\mathrm{Au}/{\mathrm{TiO}}_{2}\left\{001\right\}(●{\mathrm{OH}}_{\mathrm{adsorbed}})+\mathrm{MB}\;\to \mathrm{intermediates}+{\mathrm{CO}}_{2}+H_{2}O$$

(5) $$\mathrm{Au}/{\mathrm{TiO}}_{2}\left\{001\right\}(●{\mathrm{OH}}_{\mathrm{adsorbed}})+\mathrm{NPX}\;\to \mathrm{intermediates}+{\mathrm{CO}}_{2}+H_{2}O$$

(6) $$\mathrm{Au}/{\mathrm{TiO}}_{2}\left\{001\right\}(●{\mathrm{OH}}_{\mathrm{adsorbed}})+\mathrm{IBP}\;\to \mathrm{intermediates}+{\mathrm{CO}}_{2}+H_{2}O$$

(7) $$\mathrm{Au}/{\mathrm{TiO}}_{2}\left\{001\right\}\;(●O_{2}^{-})+\mathrm{MB}\;\to \mathrm{intermediates}+{\mathrm{CO}}_{2}+H_{2}O$$

(8) $$\mathrm{Au}/{\mathrm{TiO}}_{2}\left\{001\right\}\;(●O_{2}^{-})+\mathrm{NPX}\;\to \mathrm{intermediates}+{\mathrm{CO}}_{2}+H_{2}O$$

(9) $$\mathrm{Au}/{\mathrm{TiO}}_{2}\left\{001\right\}\;(●O_{2}^{-})+\mathrm{IBP}\;\to \mathrm{intermediates}+{\mathrm{CO}}_{2}+H_{2}O$$

## 3. Conclusions

In conclusion, we have reported on the successful modification of a facile, scalable, and cost-efficient sol–gel solution-phase route, incorporating HF and NH_4_OH as capping agents for the preparation of {001}-faceted anatase TiO_2_/Au nanosheets. The hybrid photocatalyst was examined to be photocatalytically active for the degradation of methylene blue dye, ibuprofen, and naproxen as organic contaminants in an aqueous water solution. Performance improvement of the as-synthesized NCs was observed from the visible light degradation data of an aqueous MB solution.

## 4. Materials and Methods

### 4.1. Materials and Reagents

Titanium(IV) hydroxide [Ti(OH)_4_], hydrogen fluoride (HF), methylene blue (C_16_H_13_N_3_SCl), and ammonium hydroxide (NH_4_OH) were purchased from Fluka Chemicals, Buchs, Swittzerland, Tris(triphenylphosphinegold)oxonium tetrafluoroborate from Sigma Aldrich (Burlington, MA, USA) ibuprofen, and naproxen drugs from Antibiotic a+ and KRKA d.d., respectively. Class A glassware was used throughout the experiment, and cleaned and baked for 2 h before use. 

### 4.2. Synthesis of Au/TiO_2_ Photocatalyst

The gold colloid solution was first prepared according to the procedure previously reported by Mihai et al. [32]. Briefly, 0.1452 g of Schiff base [2,3-dimethyl-1- phenyl-4-(N-2-hydroxy-3-methoxybenzaldehyde)-3-pyrazolin-5-one] and 0.0740 g of Tris (triphenylphosphinegold) oxoniumtetraflororoborate {[O(Au PPh_3_)_3_][BF_4_]} were dissolved in CH_3_CN solvent at room temperature. TiO_2_-Au NSs were synthesized via a modified sol–gel synthesis technique reported by Zhang et al. [33]. Briefly, 5 g of Ti(OH)_4_ was mixed with 15 mL HF for 150 min at 100 °C. The solution was then mixed with 15 mL of the as-synthesized gold colloid. The resulting mixture was stirred for 30 min at room temperature. The precipitation was performed using 30 mL of 1 M aqueous NH_4_OH solution and distilled water. The sample was then dried and calcined in air at 450 °C for 3 h at a 5 °C/min heating rate. The fabricated Au/TiO_2_ NSs were investigated using XRD, XPS, TEM, HR-TEM, EDS, and UV–Vis DRS.

### 4.3. X-ray Diffraction (XRD) Analysis

The crystal structure of the as-synthesized Au-TiO_2_ NCs was analyzed using a Bruker D8 Advance diffractometer (Karlsruhe, Germany) (θ-θ type) with characteristic CuKα radiation (λ = 1.5418 nm) and graphite monochromator operated at a voltage and current of 40 kV and 40 mA, respectively. The XRD pattern was recorded in the 2θ measurement range between 15° and 90° at a scan speed of 0.1°/5 s. NIST profile standards SRM 660a and SRM 1976 were used to calibrate the device and emission source profile. The qualitative analysis was carried out with the Diffracplus Basic software with the Search/Match option and the PDF-ICDD 2-2008 database. Quantitative analysis was performed with the Diffracplus TOPAS 4.1 software by the Rietveld refinement method and pseudo-Voigt function for peak matching.

### 4.4. X-ray Photolectron Spectroscopy (XPS)

The XPS spectra for the analysis of surface elements and their oxidation states were obtained using an ESCALAB XI+ Termo Scientific Photoelectron Spectrometer, Waltham, MA, U.S. Al K-α (1486.6 eV) radiation was used in the constant analyzed mode with 200 eV pass energy to plot the spectra.

### 4.5. Transmission Electron Microscopy (TEM)

To further study the structural and chemical nature of the as-synthesized sample, high resolution transmission electron microscopy (HR-TEM) was performed using an FEI Tecnai G2 F-20 TWINCryo-TEM (FEI American Company, Brno, Czech Republic) operated at an acceleration voltage of 200 kV with the magnification of 80,000 and 20,000.

### 4.6. UV–Vis Diffuse Reflectance Spectroscopy (DRS) Analysis

The spectra studies of the as-prepared materials were conducted using a UV–Vis spectrophotometer (Jasco UV–Vis V-550) (Jasco Corporation, Tokyo, Japan) in the wavelength range of 200 to 900 nm with an integrating sphere assembly. The sample was diluted with MgO (ratio 1:6) and then mechanically mixed as a reflectance standard.

### 4.7. Photocatalytic Experiment

To evaluate the photocatalytic activity of the prepared NSs, direct sunlight exposure and controlled visible light sources were employed. A Toption photochemical reactor, (TOPTION INSTRUMENT CO.,LTD., Xi’an China), equipped with a 300 W Xe lamp was used for the visible light degradation of an aqueous methylene blue solution by both pristine {001}-TiO_2_ and Au/{001}-TiO_2_ NCs. In a typical experiment, 0.025 g of the as-synthesized NCs was dispersed in 32 mg/L of the aqueous MB solution with stirring and subsequently irradiated with the photoreactor. An appropriate solution was collected at 15 min intervals to monitor the absorption spectrum using a Shimadzu 2600 UV–Vis spectrophotometer. Photocatalytic degradation efficiency of the materials was then calculated using Equation (10) below.

(10) $$\mathrm{Photodegradation}\;\mathrm{efficiency}\;\left(\%\right)=\frac{C_{0}-C_{t}}{C_{0}}\times 100\%$$

where C_0_ is the initial concentration of aqueous MB solution, and C_t_ is the concentration of MB at any specific time of visible light irradiation, respectively.

Furthermore, two cycles of photocatalytic experimental studies of the as-synthesized nanosheets for the degradation of methylene blue dye and the NSAIDs of ibuprofen and naproxen were conducted under sunlight, with a light intensity of I = 44,000 lx and a temperature of 35 °C. A UV–Vis spectrophotometer was used to monitor the reaction at 60 min intervals for 4 h in the first cycle, and at 15 min intervals for 1 h in the second cycle.

The photodegradation process in all two degradation cycles for all of the organic contaminants was examined by keeping track of the changes in the concentration in the organic content (MB, ibuprofen, and naproxen) as a function of light irradiation. Throughout the photocatalytic experiments, 0.05 g of the as-synthesized photocatalyst and 15 mL each of 4 × 10^−4^ M methylene blue, 200 mg/L ibuprofen, and 4.4 mg/L were used.

## Figures and Tables

**Table 1:** Solar-assisted photodegradation efficiencies of {001}-TiO_2_/Au NCs for cycles 1 and 2.

**Cycle 1 Photodegradation Efficiency (%)**				
Time	60 min	120 min	180 min	240 min
MB (4.10 × 10^−4^ M)	50.00	65.00	77.75	91.75
Ibuprofen (200 mg/L)	39.00	59.00	79.00	91.20
Naproxen (4.4 mg/L)	34.10	51.10	67.00	82.95
**Cycle 2 Photodegradation Efficiency (%)**				
Time	15 min	30 min	45 min	60 min
MB (4.10 × 10^−4^ M)	30.00	50.00	75.00	79.00
Ibuprofen (200 mg/L)	15.00	22.00	30.00	37.00
Naproxen (4.4 mg/L)	18.18	42.05	55.00	64.09

## Data Availability

Not applicable.
